# Testing Sex-Biased Admixture Origin of Macaque Species Using Autosomal and X-Chromosomal Genomic Sequences

**DOI:** 10.1093/gbe/evaa209

**Published:** 2020-10-12

**Authors:** Naoki Osada, Kazunari Matsudaira, Yuzuru Hamada, Suchinda Malaivijitnond

**Affiliations:** 1 Faculty of Information Science and Technology, Hokkaido University, Sapporo, Hokkaido, Japan; 2 Global Station for Big Data and Cybersecurity, GI-CoRE, Hokkaido University, Sapporo, Hokkaido, Japan; 3 Department of Biology, Faculty of Science, Chulalongkorn University, Pathumwan, Bangkok, Thailand; 4 Unit of Human Biology and Genetics, Department of Biological Sciences, Graduate School of Science, The University of Tokyo, Bunkyo-ku, Tokyo, Japan; 5 Evolutionary Morphology Section, Department of Evolution and Phylogeny, Primate Research Institute, Kyoto University, Inuyama, Aichi, Japan; 6 National Primate Research Center of Thailand, Chulalongkorn University, Saraburi Province, Thailand

**Keywords:** hybridization, sex chromosome macaque, genome

## Abstract

The role of sex-specific demography in hybridization and admixture of genetically diverged species and populations is essential to understand the origins of the genomic diversity of sexually reproducing organisms. In order to infer how sex-linked loci have been differentiated undergoing frequent hybridization and admixture, we examined 17 whole-genome sequences of seven species representing the genus *Macaca*, which shows frequent inter-specific hybridization and predominantly female philopatry. We found that hybridization and admixture were prevalent within these species. For three cases of suggested hybrid origin of species/subspecies, *Macaca arctoides*, *Macaca fascicularis ssp. aurea*, and Chinese *Macaca mulatta*, we examined the level of admixture of X chromosomes, which is less affected by male-biased migration than that of autosomes. In one case, we found that *Macaca cyclopis* and *Macaca fuscata* was genetically closer to Chinese *M. mulatta* than to the Indian *M. mulatta*, and the admixture level of Chinese *M. mulatta* and *M. fuscata*/*cyclopis* was more pronounced on the X chromosome than on autosomes. Since the mitochondrial genomes of Chinese *M. mulatta*, *M. cyclopis*, and *M. fuscata* were found to cluster together, and the mitochondrial genome of Indian *M. mulatta* is more distantly related, the observed pattern of genetic differentiation on X-chromosomal loci is consistent with the nuclear swamping hypothesis, in which strong, continuous male-biased introgression from the ancestral Chinese *M. mulatta* population to a population related to *M. fuscata* and *M. cyclopis* generated incongruencies between the genealogies of the mitochondrial and nuclear genomes.

**Significance**

1. The role of sex-biased hybridization for the origin of species is essential to understand the mechanisms of generating ecological diversity. Most of the previous studies dealing with this issue analyzed mitochondrial and Y-chromosomal data to study sex-biased migration rate, but here we focus on the genetic diversity of X chromosomes.
2. Here we investigated 17 macaque genomes. Contrasting the pattern of autosomal and X-chromosomal genetic diversity provides strong evidence of a particularly interesting demographic model, which is called the nuclear swamping model.
3. Our analysis showed strong sex-biased migration explained the observed pattern of unusual genetic differentiation among macaque species, highlighting the importance of sex-biased demographic parameters plays an important role for shaping the genomic diversity of organisms.

## Introduction

A key issue in evolutionary genetics is understanding the mechanisms by which populations are genetically differentiated and continue diverging to form separate species. Genetic differentiation is often attributed to geographical isolation, and genetically differentiated populations evolve into new, reproductively isolated, species. However, hybridization and admixture among genetically divergent populations occasionally occur, impeding further genetic differentiation. Hybridization and admixture have frequently been observed in populations of wild animals and between different species (Green et al. 2010; Osada et al. 2010; Meyer et al. 2012; Cahill et al. 2015; Fan et al. 2018).

Individuals of different sexes have shown different dispersal patterns in a wide range of sexually reproducing organisms. Sex-biased migration affects the pattern of genetic differentiation across genomes. In animals with an X-Y sex chromosome system, the X and Y chromosomes, as well as the mitochondrial genomes, can be used as markers to track sex-biased migration. The effect of migration on mitochondrial genomes depends solely upon female migration, whereas that of Y chromosomes depends solely upon male migration. Similarly, the migration of males is expected to have less impact on X chromosomes than on autosomes, because a female carries two X chromosomes but a male has only one. Genetic differentiation at these sex-linked markers, therefore, provides insights into the way in which sex-biased migration has shaped animal genomes through hybridization and admixture among populations and species (Heyer and Segurel 2010).

In order to investigate patterns of genetic differentiation across genomes during hybridization process, and to infer how sex-biased migration contributes to species divergence, we focused on macaque monkeys. The genus *Macaca* consists of 24 species distributed in the part of the Eurasian and African continents, mostly in the South, Southeast, and East Asia (Zinner 2013; Roos et al. 2014). They are classified into four to seven species groups, depending on the criteria used (Zinner 2013; Roos et al. 2019). In this study, we followed the grouping proposed by Zinner et al. (2013), and focused on four out of seven species groups: the *fascicularis* group, including *Macaca fascicularis* (the cynomolgus or long-tailed macaque); the *mulatta* group including *Macaca mulatta* (the rhesus macaque), *Macaca cyclopis* (the Taiwanese macaque), and *Macaca fuscata* (the Japanese macaque); the *sinica* group, including *Macaca sinica* (the toque macaque), *Macaca thibetana* (the Tibetan macaque), *Macaca assamensis* (the Assamese macaque), and *Macaca radiata* (the bonnet macaque); and the *arctoides* group, including *Macaca arctoides* (the stump-tailed macaque). A phylogenetic analysis using nuclear genome data showed that the *fascicularis* and *mulatta* groups and the *sinica* and *arctoides* groups are sister pairs (Fan et al. 2018).

Previous studies have revealed that ancient inter-specific gene flow has been common in macaques, not only between sister species such as *M. fascicularis* and *M. mulatta (*Osada et al. 2010; Yan et al. 2011*)* but also between different species groups, for example, *M. thibetana* in the *sinica* group and *M. mulatta* in the *mulatta* group (Fan et al. 2014). Ancient hybridization between the *arctoides* and *mulatta* groups was also supported by whole-genome sequencing analysis of *M. arctoides* (Fan et al. 2018). The divergence of the *sinica–arctoides* and *fascicularis–mulatta* groups is estimated to have occurred around 1.5–2.0 Ma, as per fossil evidence (Delson 1980), but much earlier estimates (2.8–4.9 Ma) have been obtained using nucleotide sequences (Perelman et al. 2011; Jiang et al. 2016; Matsudaira et al. 2018; Roos et al. 2019).

In some macaque species, discordant genealogies have been obtained using autosomal, mitochondrial, and Y-chromosomal loci, a situation potentially reflecting sex-biased migration patterns in the past (Tosi et al. 2000, 2002, 2003; Evans et al. 2010; Zinner et al. 2011). In our focal four species groups, three cases of incongruence between mitochondrial genealogies and the conventional taxonomic classification have been shown. 1) The mitochondrial genome of *M. arctoides* was a sister to the *mulatta*-group species in the mitochondrial phylogenetic tree (Fan et al. 2018; Roos et al. 2019); however, a genome sequencing study of *M. arctoides* showed that the *M. arctoides* nuclear genome had a strong affinity to the genomes of *sinica*-group species (Fan et al. 2018). 2) *Macaca fascicularis* ssp. *aurea* has been found to be distributed in Myanmar and Thailand, having a morphology distinct from the other nine subspecies of *M*. *fascicularis* (Fooden 1995). A genetic study using mitochondrial and Y-chromosomal sequences showed that *M. fascicularis* ssp. *aurea* clustered with the *sinica* group at the mitochondrial locus, but clustered with the *fascicularis* group at the Y-chromosomal locus (Matsudaira et al. 2018). 3) The mitochondrial sequences of *M. fuscata* and *M. cyclopis* were determined to be more similar to the Chinese *M. mulatta* than to the Indian *M. mulatta*, contradicting the conventional taxonomic classification of *M. mulatta* as a single species (Melnick et al. 1993; Matsudaira et al. 2018; Roos et al. 2019).

**Fig. 1.**
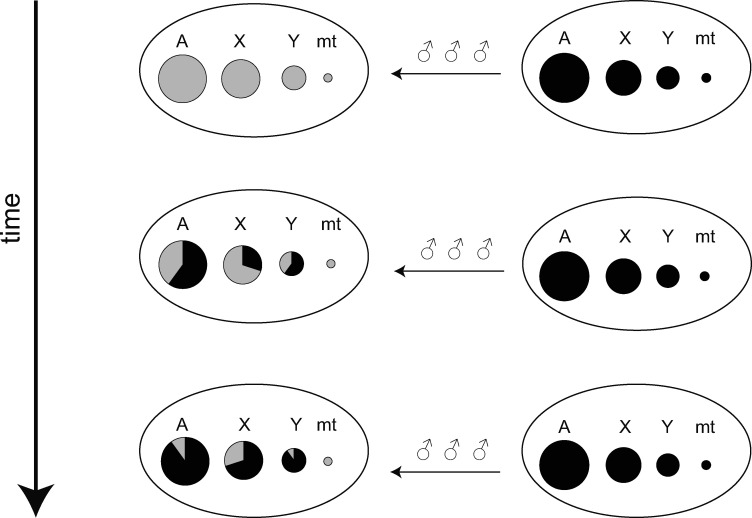
Schematic representation of the nuclear swamping hypothesis. The letters A, X, Y, and mt represent autosomal, X-chromosomal, Y-chromosomal, and mitochondrial genomes, respectively, and the circles indicate the gene pool of a population. When strong male-biased migration continues for a long time, the nuclear genomes of the recipient population are replaced by the donor alleles, whereas mitochondrial genomes retain the original (gray) genetic components. However, if the replacement is not complete, we would expect that the X chromosome would retain more original genetic components than the autosomes.

Incongruent phylogenies constructed using sex-linked loci have been often considered to be the result of male-biased migration in macaques. Because current macaque populations show predominantly female philopatry, the lineage of mitochondria is assumed to reflect the genetic lineage of the recipient population of migrants, whereas the other autosomal and Y-chromosomal genomes in the recipient population may have been “swamped” by the genomes of donor (introgressing) populations (Zinner et al. 2011, 2013). This is referred to as the nuclear swamping hypothesis. A schematic representation of the nuclear swamping hypothesis is presented in figure 1. However, in many species other than primates, introgression of mitochondrial genomes between species, so-called mitochondrial capture, is not rare (Bachtrog et al. 2006; Veale et al. 2018). Since the effective population size of the mitochondrial genome is much smaller than that of autosomal genomes, introgressed mitochondrial genomes might easily become fixed in the recipient population by genetic drift (Osada 2011). Besides, in several animal species, paternal leakage of mitochondrial genomes has been observed, particularly in cases of inter-specific hybridization (Rokas et al. 2003; Mastrantonio et al. 2019). Therefore, in order to determine the factors underlying incongruent genealogies between autosomal and sex-linked loci, a study focusing on sex-biased migration using loci other than mitochondrial locus is desirable. Genetic differentiation at the X chromosome has the potential to provide a picture with substantially higher resolution in the estimation of sex-biased migration rates, because X chromosomes are longer than mitochondria and, more importantly, recombine during female meiosis (fig. 1) (Osada et al. 2013; Evans et al. 2017; Goldberg et al. 2017).

In order to investigate how sex-biased migration affects the inter-specific hybridization of genomes, we used the above-mentioned three cases that showed incongruent species trees between mitochondrial and nuclear loci: 1) between *M. arctoides* and the *mulatta*-group species; 2) between *M. fascicularis* ssp. *aurea* and the *sinica*-group species; and 3) among the *mulatta*-group species. We clarified the phylogeny of seven macaque species using 17 whole-genome sequences, by combining newly and previously determined whole-genome sequences of macaques, and compared the level of genetic admixture at the mitochondrial genome, autosomes, and X chromosomes.

## Materials and Methods

### Sample Collection and Genome Sequencing

DNA samples of *M. fuscata* and *M. cyclopis* were obtained from the Primate Research Institute (PRI), Kyoto University. The monkeys were cared for and handled according to the guidelines established by the Institutional Animal Welfare and Animal Care Committee of PRI. They were anesthetized, and peripheral blood was obtained. The DNA samples were used for library preparation, and paired-end sequences of 101 bp were determined using HiSeq 2500 (Illumina, CA, USA). All experimental procedures were approved by the Institutional Animal Welfare and Animal Care Committee of PRI (No. 2015-138).

The three *M. fascicularis* samples were obtained from temporally caught wild animals in Thailand. The animals were anesthetized, and blood samples were withdrawn from the femoral vein. The protocol was approved by the Institutional Animal Care and Use Committee of the Faculty of Science, in accordance with the guidelines for the care and use of laboratory animals prepared by Chulalongkorn University, Protocol Review No. 1423010. DNA was extracted from the buffy coat using a standard phenol-chloroform method as described in a previous study (Bunlungsup et al. 2016). The native DNA was whole-genome amplified (WGA) and substituted to artificially synthesized DNA. WGA was conducted using the REPLI-g Mini Kit (Qiagen, Hilden, Germany) following the manufacturer’s protocol. The WGA products were purified using Wizard SV Gels and PCR Clean-Up System (Promega, WI, USA). The DNA samples were used for library preparation, and paired-end sequences of 151 bp were determined using HiSeq X (Illumina, CA, USA).

### Variant Calling

For all samples, reads were mapped to the reference genome sequence of *Papio anubis* (olive baboon, OLB; Panu_3.0, accession number: GCA_00264685.2) using the BWA-MEM algorithm (Li and Durbin 2009) with default parameter settings. Short reads potentially derived from PCR duplication were labeled using SAMBLASTER software (Faust and Hall 2014). Single nucleotide variants (SNVs) were called using HaplotypeCaller in GATK 4.0 with a prior probability of heterozygosity of 0.003 and a cutoff quality score of 30. Autosomal SNVs were jointly called across all samples. For SNV calling on X chromosomes, males and females were individually genotyped including nonvariant sites, according to the ploidy of the X chromosome. For both autosomal and X-chromosomal data, only biallelic SNVs were considered and SNVs were hard-filtered with the following parameters: QD < 2.0, FS > 60.0, SOR > 9.0, MQ < 40.0, MQRankSum < −12.5, and ReadPosRankSum < −8.0. After the filtering, the genotype data of X chromosomes from different individuals were merged using VCFtools (Danecek et al. 2011). Only the sites successfully genotyped for all 17 individuals were analyzed. In addition, we restricted the analysis to the genome positions that are highly unique in the reference genome. We used the GenMap software to calculate genome mappability (Pockrandt et al. 2020). We analyzed the site with (30, 2)-mappability = 1, which means that the 30-mer starting from the site is unique in the genome even allowing for two mismatches. Finally, pseudoautosomal regions (PARs) of X chromosome were excluded from the analysis. We downloaded the PAR sequences in humans (GRCh38) and performed homology search against the reference OLB genome. The OLB PARs were identified as chrX:1-133703 (PAR1) and chrX:143394134-143691637 (PAR2).

The runs of homozygosity (ROH) were estimated using the PLINK1.90 software using “--homogyz-kb 500” option (Purcell et al. 2007). Per-sample nucleotide diversity (the number of heterozous sites divided by the number of total genotyped sites) was calculated based on the number of heterozygous sites for each individual, calculated using PLINK1.90 “–het” option. All autosomal filtered SNVs, including coding and noncoding sites, were used for the above calculation.

### Phylogenetic Tree of Mitochondrial Genomes

We assembled mitochondrial genomes from short-read sequences using the NOVOPlasty software (Dierckxsens et al. 2016). Of the 17 samples we analyzed, 9 yielded circular mitochondrial genome assemblies. The mitochondrial genomes of Vietnamese *M. fascicularis* (CMV1) were assembled using the same method with the data obtained by Yan et al. (2011). The mitochondrial genomes of *Macaca nigra*, Bornean *Macaca nemestrina*, and *Macaca tonkeana* were assembled using the same method, with the data obtained by Evans et al. (2017). Thirty-one whole mitochondrial genomes of other macaque species and that of *P. anubis* were downloaded from the public database. The DDBJ/EMBL/GenBank accession numbers of sequences are given in supplementary table 2, Supplementary Material online. A phylogenetic tree was reconstructed using MEGA X with the maximum likelihood method (Knyaz et al. 2018). The HKY substitution and G + I (gamma + invariant sites) rate models were used.

### Analysis of Species Phylogeny and Genetic Structure

To construct a neighbor-joining tree and neighbor-net network, all pairwise identity-by-decent (IBD) distances were calculated using the PLINK1.90 with “–distance 1-ibs” option. All filtered sites on autosomes including monomorphic sites were considered. The neighbor-joining tree and neighbor-net network were constructed using the phangorn package in R (Schliep 2011) using the distance matrix generated by PLINK. Principal component analysis (PCA) was performed using smartpca software with a default parameter setting (Patterson et al. 2006). TreeMix software was run using the block size of 1000 SNVs (Pickrell and Pritchard 2012).

### Analysis of Species Admixture

We used AdmixTools to compute *f*_3_ and *f*_4_ statistics (Patterson et al. 2012), using 5 Mb as a unit of block jackknife. We describe the configuration of outgroup *f*_3_ statistics as *f*_3_(A, B; C), where the sample C is the outgroup species (OLB). The larger *f*_3_(A, B; C) indicates that the sample A and B have a larger amount of shared genetic drift, which means A and B are genetically close. We represent the configuration of *f*_4_ statistics as *f*_4_(A, B; C, D), where A–D represent the names of the populations to which the samples belong. We fixed the species A to OLB, since we assumed that OLB has not experienced any recent gene flow with our macaque populations. As *f*_4_ statistics are equivalent to *D* statistics, the statistics do not deviate from 0 when the genetic relationship between the four species/populations is represented as a tree structure, indicating no gene flow between species/populations (Patterson et al. 2012). When gene flow occurs between B and D, the *f*_4_ statistics become positive. When gene flow occurs between B and C, the *f*_4_ statistics become negative.

In order to reconstruct an admixture graph, we used qpGraph software (Patterson et al. 2012). Starting from the species phylogeny inferred from the neighbor-joining tree, we added migration edge one by one to resolve the incongruence with the highest absolute value for Z score. The deviation of the final model was not significant: *f*_4_ statistics of 0.0095 and Z score of 0.520.

## Results

### Whole-Genome Sequencing and Genetic Diversity in Populations

This study has performed whole-genome sequencing of five macaque samples: one female *M. fascicularis* ssp. *aurea (*cynomolgus macaque aurea 1, CMA1*)*, one male *M. fascicularis* ssp. *aurea (*cynomolgus macaque aurea *2*, CMA2*)*, one male *M. fascicularis* ssp. *fascicularis* from Southern Thailand *(*cynomolgus macaque in Thailand 1, CMT1*)*, one male *M. fuscata (*Japanese macaque 1, JPM1*)*, and one male *M. cyclopis* (Taiwanese macaque 1, TWM1) (supplementary table 1, Supplementary Material online). We also obtained short-read sequences of additional 12 samples from previous studies (supplementary table 2, Supplementary Material online): three Chinese *M. mulatta* (rhesus macaque in China 1–3, RMC1–3), two Indian *M. mulatta* (rhesus macaque in India 1–2, RMI1–2), three Mauritian *M. fascicularis* ssp. *fascicularis* (cynomolgus macaque in Mauritius 1–3, CMM1–3), one Vietnamese *M. fascicularis* ssp. *fascicularis* (cynomolgus macaque in Vietnam 1, CMV1), one Chinese *M. assamensis* ssp. *assamensis* (Assamese macaque 1, ASM1), one *M. thibetana* (Tibetan macaque 1, TIM1), and one *M. arctoides* from China (stump-tailed macaque 1, STM1). The abbreviations of the sample names are summarized in table 1. These symbols are used to represent the names of the samples.

**Table 1:** Summary of Whole-Genome Sequencing

Sample	Symbol	Coverage	Per-sample Nucleotide Diversity	References
*M. fascicularis* spp. *fascicularis* (Thailand)	CMT1	39.8	0.0034	This study
*M. fascicularis* spp. *fascicularis* (Vietnam)	CMV1	32.0	0.0029	Yan et al. (2011)
*M. fascicularis* spp. *fascicularis* (Mauritius)	CMM1	36.8	0.0025	Ericsen et al. (2014)
*M. fascicularis* spp. *fascicularis* (Mauritius)	CMM2	35.5	0.0024	Ericsen et al. (2014)
*M. fascicularis* spp. *fascicularis* (Mauritius)	CMM3	39.6	0.0026	Ericsen et al. (2014)
*M. fascicularis* ssp. *aurea*	CMA1	45.7	0.0012	This study
*M. fascicularis* ssp. *aurea*	CMA2	41.7	0.0029	This study
*M. mulatta* (China)	RMC1	34.4	0.0025	Xue et al. (2016)
*M. mulatta* (China)	RMC2	23.5	0.0024	Xue et al. (2016)
*M. mulatta* (China)	RMC3	29.3	0.0024	Xue et al. (2016)
*M. mulatta* (India)	RMI1	40.2	0.0022	Xue et al. (2016)
*M. mulatta* (India)	RMI2	47.6	0.0023	Xue et al. (2016)
*M. fuscata*	JPM1	27.5	0.0010	This study
*M. cyclopis*	TWM1	40.0	0.0017	This study
*M. thibetana*	TIM1	38.1	0.0009	Fan et al. (2014)
*M. assamensis*	ASM1	50.8	0.0028	Fan et al. (2018)
*M. arctoides*	STM1	33.2	0.0017	Fan et al. (2018)

Because we focused on admixture between different species groups, the reads were mapped to the reference genome sequence of OLB, which should be equally distant from all the analyzed samples, in order to avoid mapping bias due to short reads. The mapping rate to the OLB genome was greater than 99% except for one sample, RMC2 (supplementary fig. 1, Supplementary Material online). After the filtering, we obtained 66,881,813 autosomal and 1,797,069 X-chromosomal biallelic SNVs successfully called in all samples. The numbers of SNVs before and after filtering are presented in supplementary table 3, Supplementary Material online.

The coverage of the genomes and the per-sample nucleotide diversity on autosomes (the number of different nucleotides per base-pair between paternal and maternal chromosomes) are summarized in table 1. CMA1, JPM1, and TIM1 showed very low levels of genetic diversity (0.0009–0.0012), whereas CMA2, CMT1, CMV1, and ASM1 were determined to have the highest levels of genetic diversity (0.0028–0.0034). In order to evaluate the low observed genetic diversity of CMA1, JPM1, and TIM1, we calculated the ROH regions greater than 500-kb length for each sample (supplementary table 4, Supplementary Material online). Although the per-sample nucleotide diversity of CMA1 was higher than that of JPM1, the number of ROH regions in CMA1 was noticeably higher than that in JPM1 (892 vs. 265), suggesting that the low genetic diversity of CMA1 may be due to a recent inbreeding effect. We also plot per-sample nucleotide diversity and the length of total ROH regions in supplementary figure 2, Supplementary Material online. Although the per-sample nucleotide diversity values and ROH lengths were highly correlated, CMA1 showed unusually long ROH regions in the genome.

### Species Phylogeny

**Fig. 2.**
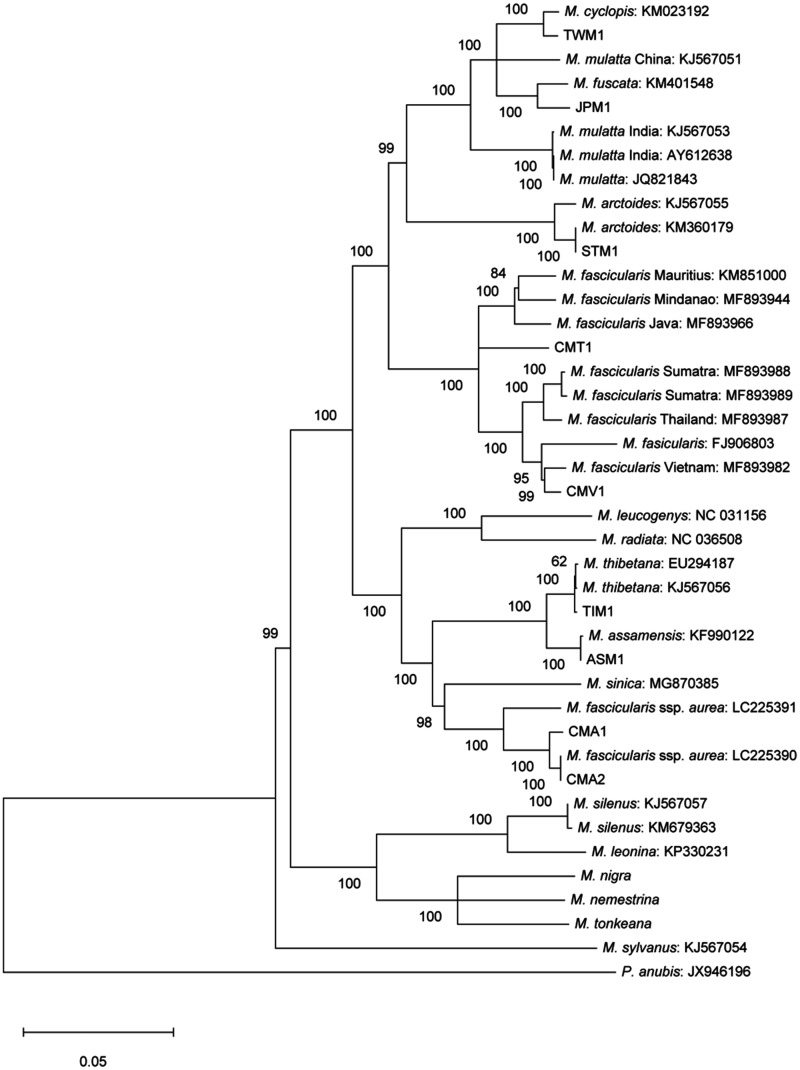
Mitochondrial genealogy, including publicly available mitochondrial genome sequences. Bootstrap percentile values are shown near the nodes.

To examine the phylogenetic relationships among the macaques, we first constructed a tree using mitochondrial genomes (fig. 2). Complete mitochondrial genome sequences were assembled for the five newly sequenced samples (CMT1, JPM1, TWM1, CMA1, and CMA2) and four additional samples (CMV1, ASM1, STM1, and TIM1). Additional 31 mitochondrial genomes of macaques were directly downloaded from the public database or assembled using short-read sequences in the public database (supplementary table 5, Supplementary Material online). As shown in figure 2, at the mitochondrial locus, *M. arctoides* clustered with the *mulatta* group, whereas *M. fascicularis* ssp. *aurea* clustered with the *sinica* group. The tree also indicated that *M. mulatta* samples were paraphyletic; the Indian *M. mulatta* samples were considered an outgroup for the Chinese *M. mulatta*, *M. fuscata*, and *M. cyclopis* samples. The result of our study is largely consistent with the result of the previous one (Roos et al. 2019).

**Fig. 3.**
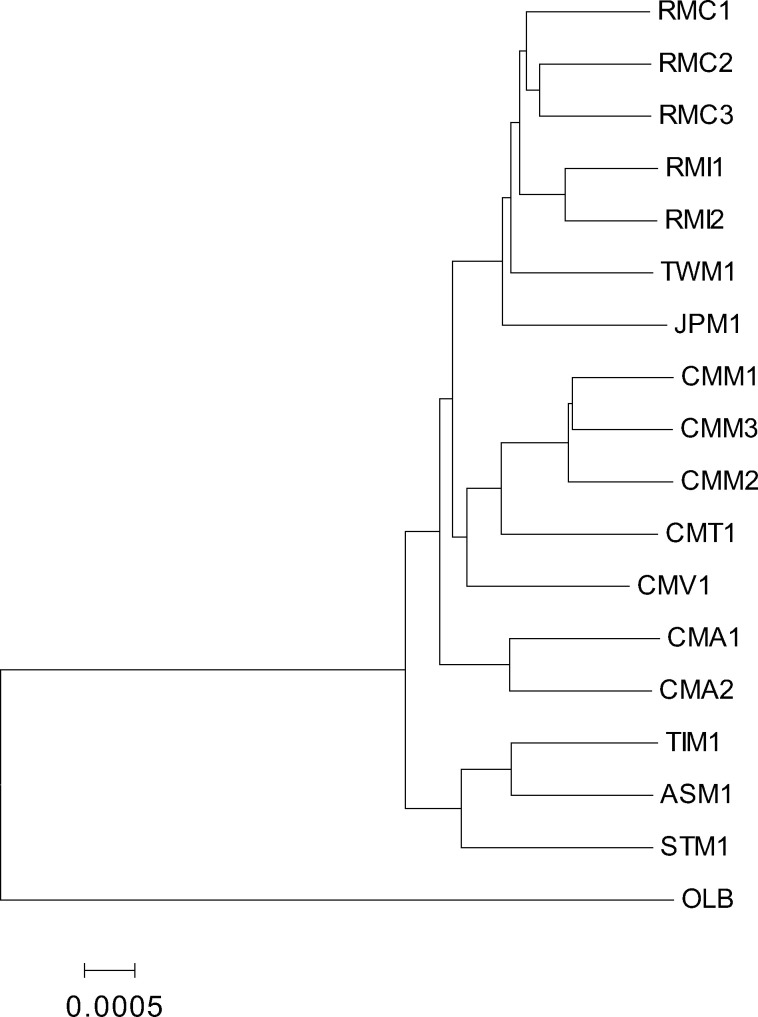
Neighbor-joining tree constructed using the IBD distances between nuclear genomes.

Next, we constructed a neighbor-joining tree using the genetic distances between autosomes with the draft genome sequence of OLB as an outgroup (fig. 3). The tree showed the sister relationship between the *sinica* and *arctoides* groups, and between the *fascicularis* and *mulatta* groups, as presented in previous studies (Perelman et al. 2011; Fan et al. 2014, 2018). We also reconstructed a tree using the TreeMix software (Pickrell and Pritchard 2012) and a graph using the neighbor-net method (Bryant and Moulton 2004). These results were found to be essentially the same as the neighbor-joining tree (supplementary figs. 3 and 4, Supplementary Material online) but showed a signature of gene flow between species. We also performed PCA among samples (supplementary fig. 5, Supplementary Material online). The major cluster which corresponds to the *fascicularis* group, *mulatta* group, and *sinica*-*arctoides* group were observed on the plot using the first and second principal components. The plot showed that CMA1 and CMA2 were closer to the *sinica–arctoides* group than the other *M*. *fascicularis*. The plot also showed that Vietnamese *M*. *fascicularis* (CMV) was closer to the *mulatta*-group than the other *M*. *fascicularis*.

**Fig. 4.**
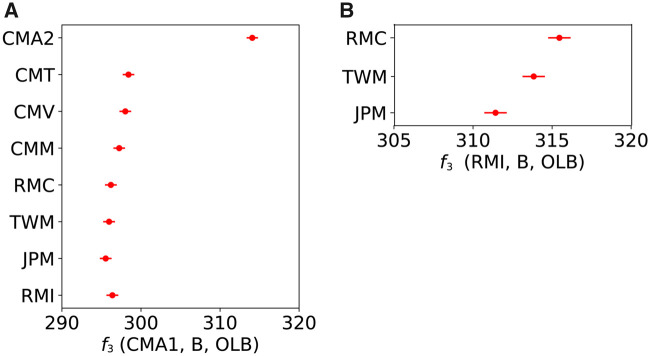
F_3_ statistics. (*A*) *f*_3_(CMA1, B, OLB), where population B is shown on the left side of the panel. Large values mean that B is genetically close to CMA1. (*B*) *f*_3_(RMI, B, OLB), where population B is shown on the left side of the panel. Large values mean that B is genetically close to RMI.

As shown in figure 3, CMA1 and CMA2 were placed at the root of the *fascicularis–mulatta* clades, which is contradicting to the conventional taxonomic classification of *M. fascicularis* ssp. *aurea*. In order to statistically evaluate the genetic relationship between CMA1/CMA2 and other *fascicularis* and *mulatta* group species, we evaluated *f*_3_ statistics (or outgroup *f*_3_ statistics) using the OLB genome as the outgroup. In the following analyses based on population genetics theory, we use three-letter symbols to represent the names of the populations to which samples belong. For example, RMC represents the population of which RMC1, RMC2, and RMC3 are members. Although we have only one individual for each of the CMT, CMV, TWM, JPM, STM, TIM, and ASM populations, we can assume that the genotype of the sampled individual reflects the allele frequency of SNVs in each population. However, we treated samples CMA1 and CMA2 as if they were in different populations, since we do not have any priori assumptions about their population structure. The *f*_3_ statistics showed that CMA1 and CMA2 were generally closer to CMT and CMV than to CMM or the populations in the *mulatta* group (fig. 4*A*). For example, the outgroup *f*_3_ of CMA1 and CMT was significantly greater than that of CMA1 and RMC (*P *=* *0.017, Welch’s *t*-test), supporting that CMA1 and CMA2 were *fascicularis*-like, rather than *mulatta*-like, populations.

The phylogenetic relationship among the three species of the *mulatta* group—*M. mulatta*, *M. fuscata*, and *M. cyclopis*—has not previously been examined at the whole-genome level. In the neighbor-joining tree shown in figure 3, the Chinese *M. mulatta* samples clustered with the Indian *M. mulatta* samples, and *M. mulatta* was monophyletic and sister to *M. cyclopis*. However, the branching pattern of the tree may be distorted by gene flow between species/populations. The *f*_3_ statistics agreed with the pattern in the neighbor-joining tree and indicated that *M. mulatta* and *M. cyclopis* were the sister pair (fig. 4*B*).

### General Picture of Admixture between Species/Populations

We have examined the presence of gene flow between species/populations using *f*_4_ statistics (see Materials and Methods). Hereafter, we use the symbol *f*_4__A_ to represent *f*_4_ statistics computed using an autosomal genome. We computed *f*_4__A_(OLB, B; C, D) by choosing B, C, and D for all 220 combinations of sampled populations. Of these, 160 combinations were found to be significant, according to the *f*_4_ statistics (*P *<* *0.0001 after Bonferroni correction) in the best-fitted tree topologies (supplementary table 6, Supplementary Material online). These results indicate that the phylogenetic relationships of macaques are highly reticulate, as suggested by Fan et al. (2018).

The deviation from the tree structure was found to be particularly strong when the configuration included STM, CMA1, CMA2, CMT, and CMV. The all populations in *fascicularis–mulatta* group showed a stronger affinity to STM than to the populations in the *sinica* group. For example, *f*_4__A_(OLB, RMC; STM, TIM) was strongly negative (*f*_4__A_, −0.0019; Z score, −46.3). Similarly, the populations in the *sinica* group were significantly closer to CMA1 and CMA2 than to the populations in the other *fascicularis–mulatta* group. Besides, CMV and CMT were more closely related to RMC than to RMI.

### Regional Heterogeneity of Genetic Differentiation on the X Chromosomes

Before estimating the level of admixture on the X chromosome, we investigated the regional heterogeneity of genetic differentiation on the X chromosome. We computed *f*_4_ statistics for each SNV across the non-PAR of the X chromosome. We found that an approximately 10-Mb length region proximal to PAR1 boundary showed an unusual pattern of differentiation. An example of the *f*_4_(OLB, ASM; RMI, CMV) across the non-PAR of the X chromosome is shown in supplementary figure 6, Supplementary Material online. The *f*_4_ values near the PAR1 boundary were observed to be strongly negative and showed unusually high variance. This pattern was consistently observed in comparisons involving few samples. Although we could not identify the reason for this unusual pattern of differentiation, we excluded the region from further analysis. Hereafter, we designate the *f*_4_ statistics on the X chromosome excluding the PARs and the 10-Mb region proximal to PAR1 boundary as *f*_4__X_.

### Contrasting the Patterns of Admixture on Autosomes and the X Chromosome

**Fig. 5.**
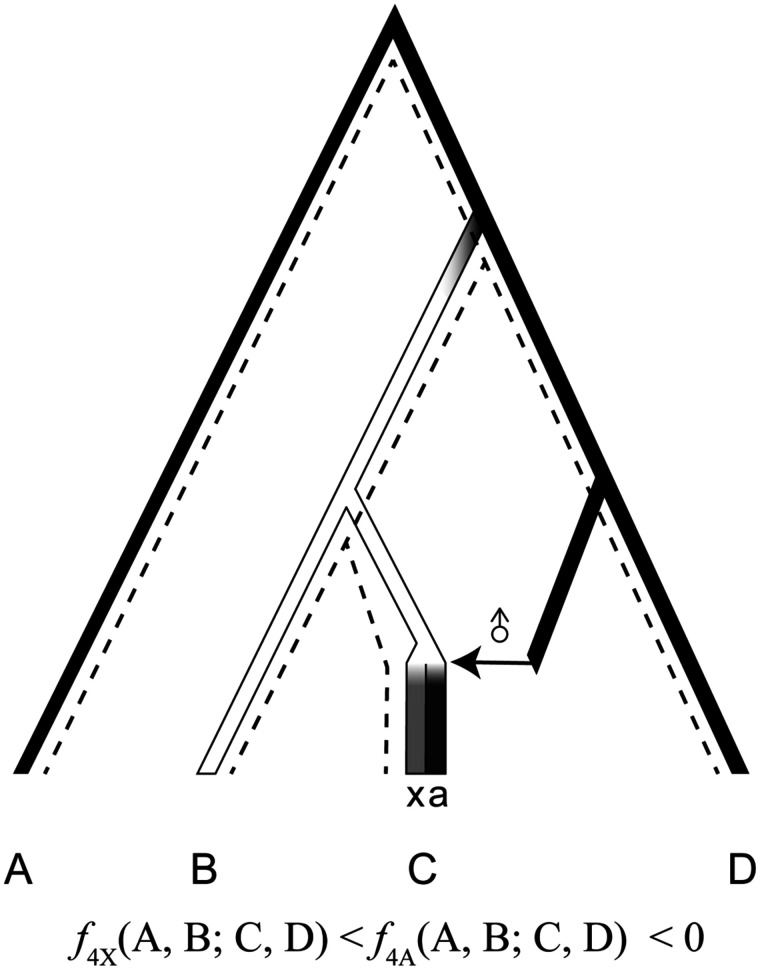
Schematic illustration of testing the nuclear swamping model using *f*_4_ statistics. We analyze four populations, A–D, where A is the outgroup population. The tick bars represent the history of nuclear genomes, and the dashed lines show mitochondrial genealogy. In this scenario, the common ancestors of B and C split from the lineage of D, and genetic drift has changed allele frequencies in populations (represented by black and white colors). After the divergence of B and C, we assume strong and continuous male-biased migration events from the sister population of D (the horizontal arrow). Finally, the autosomal (and Y-chromosomal) genome was almost replaced by the one from the donor population (the dark gray bar over “a”). On the other hand, the replacement of X-chromosomal genome would be weak so that the X chromosomes retained original genetic variation (the light gray bar over “x”). When the replacement is not complete, *f*_4__A_(A, B; C, D) becomes negative, but the deviation would be milder than *f*_4__X_(A, B; C, D).

We compared the pattern of genetic differentiation between autosomes and X chromosomes. If incongruent genealogies between mitochondrial and autosomal loci, as observed above, are due to a nuclear swamping process, we expect that X chromosomes are less affected by introgression than autosomes. To test the bias, we statistically compared *f*_4_ values between autosomes and X chromosomes under the situation presented in figure 5. We expect that *f*_4_ statistics becomes more strongly negative on X chromosomes than on the autosomes.

**Fig. 6.**
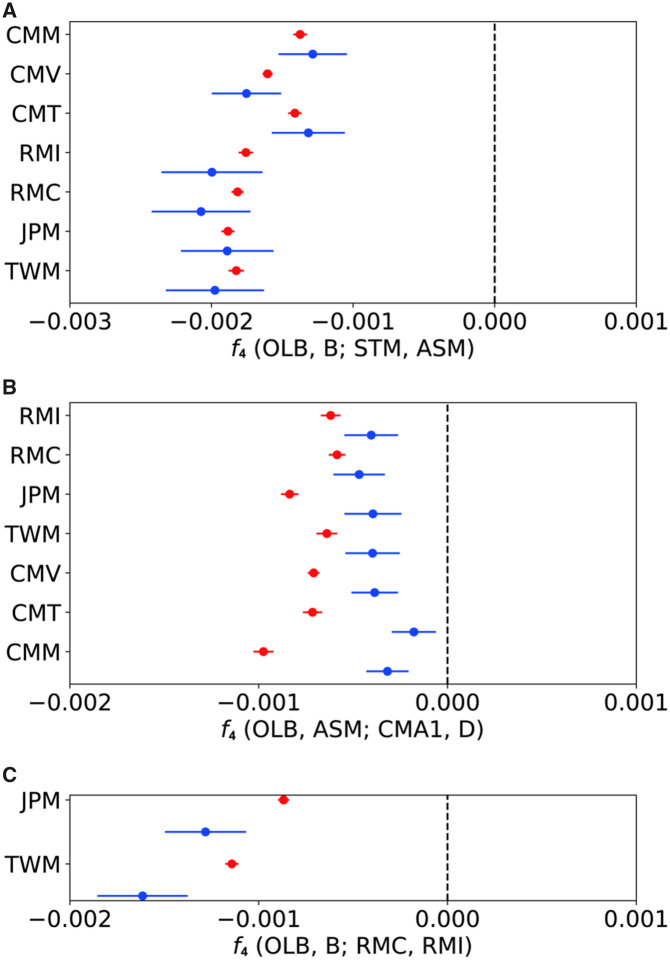
The values of *f*_4_ statistics on autosomes (red) and the X chromosome (blue). The names of the target species/population are shown on the left side of the panel. (*A*) *f*_4_(OLB, B; STM, ASM). Negative *f*_4_ values indicate that B is more closely related to STM than to ASM. (*B*) *f*_4_(OLB, ASM; CMA1, D). Negative *f*_4_ values indicates that ASM is more closely related to CMA1 than to D. (*C*) *f*_4_(OLB, B; RMC, RMI). Negative *f*_4_ values indicate that B is more closely related to RMC than to RMI.

We first examined the level of admixture between STM and the *fascicularis–mulatta*-group populations. The values of *f*_4__A_(OLB, B; STM, ASM), where B represents a population in the *fascicularis* or *mulatta* group, were significantly biased in the negative direction (fig. 6*A*). The *f*_4__A_ values with the *mulatta* group were generally lower than those of the *fascicularis* group, indicating a strong affinity of STM to the *mulatta* group. If the *mulatta*-like mitochondrial genomes of STM originated via nuclear swamping, we could expect that the X chromosomes of STM would be more closely related to those of the *mulatta* group and that *f*_4__X_(OLB, B; STM, ASM) would be lower than *f*_4__A_(OLB, B; STM, ASM). The results showed that the *f*_4__X_ had very large variance across the chromosome and were not significantly different from *f*_4__A_. In addition, there was no consistent trend in the differences between the *f*_4__X_ and *f*_4__A,_ as shown in figure 6*A*.

We next compared *f*_4__A_ and *f*_4__X_ including CMA1 and CMA2. We computed the *f*_4_(OLB, ASM; CMA1/2, D), where D represents a population in the *fascicularis* or *mulatta* group other than CMA1 and CMA2. The *f*_4__A_ values were observed to be strongly negative, indicating that ASM was significantly more closely related to CMA1 and CMA2 than to the other *fascicularis-* and *mulatta*-group populations (fig. 6*B*), which coincides with the observation that the mitochondrial genomes of CMA1 and CMA2 clustered with those of the *sinica* group (fig. 2). Similar to the previous analysis, if the *sinica*-like mitochondrial genomes of CMA1 and CMA2 were the consequence of nuclear swamping, we would expect that the X chromosomes of CMA1 and CMA2 would be more closely related to those of the *sinica* group than to the autosomes, resulting in *f*_4__X_ being lower than *f*_4__A_. We observed that the *f*_4__X_ values were consistently higher than the *f*_4__A_ values, which was opposite direction from the expectation. Again, the results did not consistently support the nuclear swamping hypothesis.

We finally looked at the *f*_4_ statistics to detect admixture among the species/populations in the *mulatta* group. We computed *f*_4__A_ and *f*_4__X_ values for *f*_4_(OLB, JPM; RMC, RMI) and *f*_4_(OLB, TWM; RMC, RMI). In both cases, the *f*_4__A_ and *f*_4__X_ values were significantly negative, indicating that JPM and TWM were more closely related to RMC than to RMI (fig. 6*C*). Since *M. fuscata* and *M. cyclopis* do not form a sister species pair (fig. 3), this result indicates that there were at least two rounds of past admixture events among the ancestral populations of Chinese *M. mulatta*, *M. fuscata*, and *M. cyclopis.* This pattern of admixture is consistent with the mitochondrial phylogeny, in which individuals of Chinese *M. mulatta* clustered with JPM1 and TWM1. We also found that *f*_4__X_ was significantly lower than *f*_4__A_ (*P *=* *0.034 for JPM and *P *=* *0.030 for TWM, Welch’s *t*-test), which shows that RMC were more similar to JPM and TWM on the X chromosomes than on autosomes, a pattern expected under the nuclear swamping hypothesis.

**Fig. 7.**
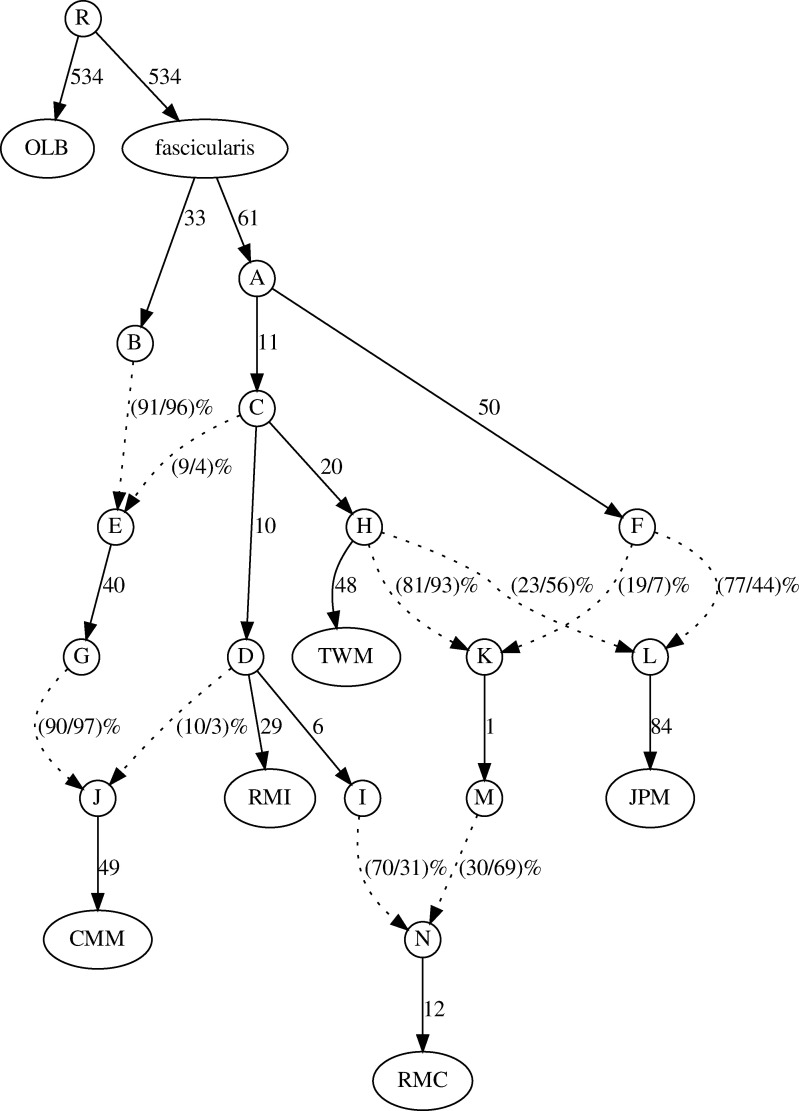
Admixture graph of the *mulatta*-group species and CMM. OLB was used as the outgroup. The numbers along the branches (solid lines) indicate drift parameters estimated using autosomes. The dashed lines represent admixture events, and the numbers in parenthesis show the admixed fraction (%) estimated using autosomes (left) and X chromosomes (right). In this figure, the establishment of the RMC population is explained by two admixture events. 1) The admixture of populations related to the JPM lineage (node F) and a population related to TWM lineage (node H) created a hybrid population (node K). 2) The admixture of the hybrid lineage (node M) and a population diverged from RMI (node I) generated the ancestral population of RMC (node N). Note that the admixture fraction of node N from node I is much larger on autosomes than on X chromosomes (70% and 30% on autosomes and X chromosomes, respectively). This admixture graph did not show any statistically significant deviation in thems of *f*_4_ statistics.

To support these observations, we constructed an admixture graph using autosomal data. The admixture graph suggested a complex admixture history of the *mulatta* group (fig. 7). Although the reconstructed admixture graph is only one of the good past demographic models that explains the data, the admixture graph was concordant with the hypothesis of two-round hybridization for the origin of Chinese *M. mulatta* inferred by the *f*_4_ statistics. Based on this model, the populations that diverged from the lineages of *M. fuscata* and *M. cyclopis* generated a hybrid population (node K in fig. 7), and later, this hybrid population admixed with the ancestral population of Chinese *M. mulatta* (node I in fig. 7).

We assume that the admixture graph inferred from the autosomal data reflects the past demographic events among species reasonably accurately and estimated the admixed fraction on the X chromosome by fixing the admixture graph (fig. 7). In general, the level of admixture on the X chromosomes was lower than that on autosomes. However, the admixed fraction from the ghost population (node M in fig. 7) in the RMC genomes on the X chromosomes was 69%, which was much higher than that on autosomes (30%). This result is consistent with the pattern observed in the analysis of the *f*_4_ statistics, and this also supports the nuclear swamping hypothesis, which posits that strong, continuous male-biased migration from the ancestral Chinese *M. mulatta* population (node I in fig. 7) to the ghost population diverged from *M. fuscata* and *M. cyclopis* (node M in fig. 7) generated incongruencies between the genealogies of the mitochondrial and autosomal genomes.

## Discussion

In this study, we examined 17 macaque genomes to investigate their evolutionary history. Comparing the pattern of genetic differentiation between the autosomal and X-chromosomal genomes is the primary focus of this study, in order to quantify the effect of sex-biased migration, which is universally observed in sexually reproducing organisms. The number of samples per species in this study was limited, and we mostly neglected the genetic structure within species. In particular, *M. mulatta* and *M. fascicularis* are widely distributed across Southeast and East Asia, and they showed detectable levels of genetic structure within the species (Bunlungsup et al. 2017; Liu et al. 2018). Therefore, a more detailed and comprehensive evolutionary history of macaques should be performed using species-wide genome sequences in future studies. Despite this limitation, our analysis including five newly sequenced genomes revealed many novel aspects of macaque evolutionary history.

The genetic relationship of *M. fascicularis* ssp. *aurea* to other species has only been recently studied using mitochondrial and Y-chromosomal genomes (Matsudaira et al. 2018). It was reported that *M. fascicularis* ssp. *aurea* used stone tools for foraging (Carpenter 1887; Malaivijitnond et al. 2007), which is a unique behavior to the subspecies. Our genome-wide analysis suggested a highly complex scenario for the origin of the subspecies. Strong admixture was detected between *M. fascicularis* ssp. *aurea* and the *sinica* group; this implies that *M. fascicularis* ssp. *aurea* originated from the ancient hybridization with a population related to *sinica*-group species. *f*_3_ statistics indicated that CMA1 and CMA2 are most closely related to the other *M. fascicularis* (fig. 4*A*). The comparison between *f*_4_ statistics on autosomes and X chromosomes did not supported the nuclear swamping hypothesis. Surprisingly, we found that the PCA plot using X-chromosomal data showed that the X chromosomes of CMA1 and CMA2 had very different genetic features (represented in the second principal component) compared with the other samples (supplementary fig. 7, Supplementary Material online), indicating that the X chromosomes of *M. fascicularis* ssp. *aurea* may have originated from unknown species that were not sampled in our study. Given the highly complex genetic feature of *M. fascicularis* ssp. *aurea*, it is difficult to determine whether the *M. fascicularis* ssp. *aurea* lineage branched after or before the split of the *fascicularis* and *mulatta* groups at this moment.

Our results showed that the genomic features of *M. fascicularis* ssp. *aurea* are unique among the *M. fascicularis* samples. CMA1 had very low genetic diversity compared with the other *M. fascicularis* individuals in this study. The large number of ROH in CMA1 (supplementary fig. 2 and table 4, Supplementary Material online) implies that the reduction of genetic diversity may be due to recent inbreeding. Indeed, CMA1 was sampled from a population living on a small island, and the population size remained the same for decades.

The genome-wide phylogenetic relationship among species in the *mulatta* group was also clarified in this study. Using approximately 8 Mb of autosomal sequence data, Perelman et al. (2011) reported a sister relationship between *M. mulatta* and *M. fuscata*. However, our analysis showed that *M. mulatta* is closer to *M. cyclopis* than to *M. fuscata* at autosomal loci (figs. 3 and 6*C*), a finding which is concordant with those of a study using isozymes by Melnick and Kidd (1985). In addition, *f*_4_ statistics showed a high level of admixture between Chinese *M. mulatta* and *M. fuscata*/*cyclopis*. Since *M. fuscata* and *M. cyclopis* were found to not form a sister pair, there have been multiple admixture events among the *mulatta*-group species. In order to provide deeper insights into the evolutionary history of the *mulatta*-group species, we reanalyzed population genomics data produced by Liu et al. (2018) from the Chinese *M. mulatta*, including five subspecies: *M. m.* ssp. *tcheliensis*, *M. m.* ssp. *littoralis*, *M. m.* ssp. *brevicaudus*, *M. m.* ssp. *lasiotis*, and *M. m.* ssp. *mulatta*. One individual of each subspecies was added to our data set (supplementary table 7, Supplementary Material online). Although all the subspecies showed strong signatures of admixture with *M. fuscata* and *M. cyclopis*, significant heterogeneity in the relatedness of Chinese *M. mulatta* to *M. cyclopis* and *M. fuscata* was found; *f*_4_ statistics showed that the subspecies *M. m.* ssp. *tcheliensis* from North China and *M. m.* ssp. *mulatta* from Yunnan were more dissimilar to *M. cyclopis* and *M. fuscata* than the other subspecies (supplementary fig. 8, Supplementary Material online). The two subspecies currently inhabit peripheral regions to the range of *M. mulatta.* Since *M. m.* ssp. *tcheliensis* would be the latest derived subspecies (Liu et al. 2018), the process of admixture would have continued in multiple regions around Southern China.

In an evolutionary history proposed by Delson (1980), the most recent common ancestors of *M. mulatta* lived around Eastern India or Myanmar and split to form the Indian and Chinese populations. Using genome-wide polymorphism data, Hernandez et al. (2007) estimated the split to occur around the Middle to Late Pleistocene boundary (162 kya). After the split, the Chinese population expanded eastward and northward as their population size increased. Both mitochondrial and X-chromosomal patterns were explained by the hypothesis that strong male-biased migration from the ancestral Chinese *M. mulatta* population toward the ghost population related to *M. fuscata* and *M. cyclopis* occurred after the split between the Chinese and Indian populations. This scenario is also supported by the reconstructed admixture graph (fig. 7). We show the hypothetical evolutionary history of the *mulatta*-group species in supplementary figure 9, Supplementary Material online.

In this study, we observed significant levels of gene flow among many pairs of species. Of the 220 possible configurations of *f*_4_ statistics, 160 have showed deviation from a simple phylogenetic tree (*P *<* *0.0001). The reason for the large number of deviant comparisons might be that the *f*_4_ test is too sensitive. We tested the deviation of *f*_4__A_(OLB, STM; C, D), where C and D represent individuals from the same populations, and estimated the degree of deviation from treeness (STM was selected with arbitrary decision). Among seven possible comparisons, only one pairs *f*_4__A_(OLB, STM; RMI1, RMI2) showed slight deviation from the null model (*f*_4_ = −0.0001, Z = −6.93). We cannot confirm whether these deviations are due to technical artifacts or to hidden population structure. However, most of the observed degrees of deviation in our main analysis were much higher than these values; 62 out of the 220 comparisons had a Z score greater than 20 or smaller than −20, indicating that most of the inferred admixture events accurately reflect past demographic events.

For *M. arctoides* and *M. fascicularis* ssp*. aurea*, we did not find evidence supporting the nuclear swamping hypothesis for their origination. Given the significant gene flow among species, the evolutionary history of the *fascicularis* and *sinica* groups would be extremely complicated and may have blurred the signature of the past sex-biased migration. Alternatively, the incongruence between autosomal and mitochondrial genealogies could be explained by the introgression of mitochondrial genomes. Studies with larger sample sizes may be conducted to answer the question in the near future.

We should also note that the different patterns of genetic differentiation between autosomal and X-chromosomal loci expected under nuclear swamping are also explained by strong natural selection against the introgression of X chromosomes. These factors are not mutually exclusive, and their relative importance should be examined using multiple sources of evidence. In the case of Chinese *M. mulatta*, if we assume that the mitochondrial locus is evolutionarily neutral, the observed pattern provides evidence supporting the nuclear swamping hypothesis. However, several studies have suggested that the introgression of mitochondrial genomes might be deleterious because they may disrupt the interactions between nuclear and mitochondrial alleles, a phenomenon which is referred to as mitonuclear incompatibility (Osada and Akashi 2012; Hill 2020). In such a scenario, the patterns expected under male-biased migration and selection against X-chromosomal/mitochondrial introgression would be indistinguishable. Therefore, in order to understand the factors shaping the diversity of genomes and elucidate the natural history of organisms, population genomic studies on a wide range of organisms with different sex-determination systems and different migration patterns are needed.

## Conclusions

In this study, we investigated the complex evolutionary history of macaques with rampant gene flow among species, using a whole-genome sequence data set. As shown in previous studies, incongruencies in genealogical relationships between nuclear and mitochondrial genomes were observed among species. We showed that comparing the pattern of admixture between autosomal and X-chromosomal loci is a potentially valuable approach to statistically evaluate the power of sex-biased migration in shaping the pattern of genome evolution. We detected a statistically significant difference in admixture levels between these loci, which could be explained by strong male-biased migration in one of the three cases tested. Comparisons between an autosomal and an X-chromosomal evolutionary pattern using a larger data set in other species would reveal a more detailed evolutionary history in future studies.

## Supplementary Material

Supplementary data are available at *Genome Biology and Evolution* online.

## Supplementary Material

evaa209_Supplementary_Data
