# Supplementary material for: Testing Sex-Biased Admixture Origin of Macaque Species Using Autosomal and X-Chromosomal Genomic Sequences
Source: Genome Biol Evol. 2020 Oct 12;13(1):evaa209. doi: 10.1093/gbe/evaa209 (PMC8631084; doi:10.1093/gbe/evaa209)
Supplement: evaa209_Supplementary_Data [file evaa209_Supplementary_Data.zip › supplementaryFigures_200902.docx]

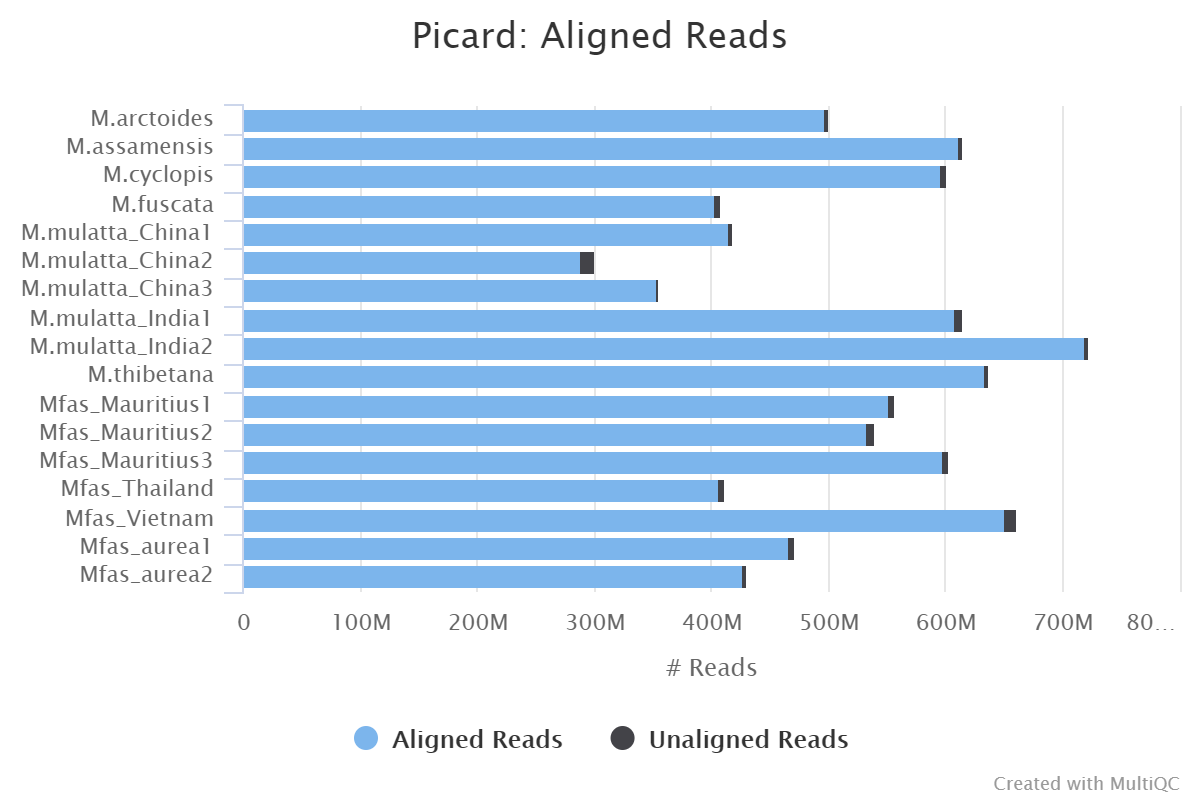


**Supplementary Figure 1**

Numbers of the total and mapped reads, generated using the MultiQC software (Ewels et al. 2016).


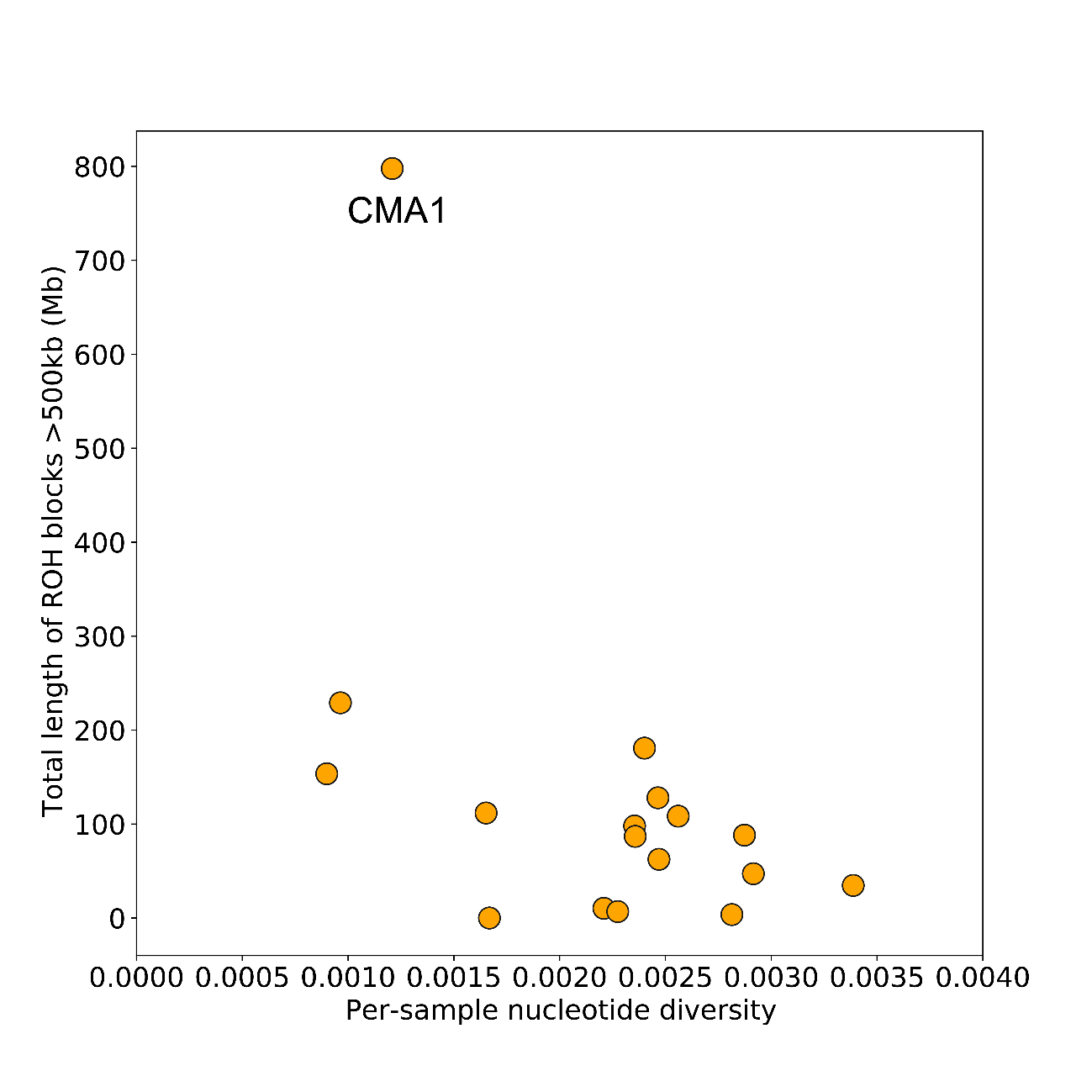


**Supplementary Figure 2**

Inbreeding coefficient (F_IS_) and total length of ROH (>500 kb) blocks in the genome for each sample are plotted. CMA1 represents the *M. fascicularis* ssp. *aurea* sample from Wat Paknam Pracharangsarith, Ranong, Thailand. The detailed information is presented in Supplementary Table 4.


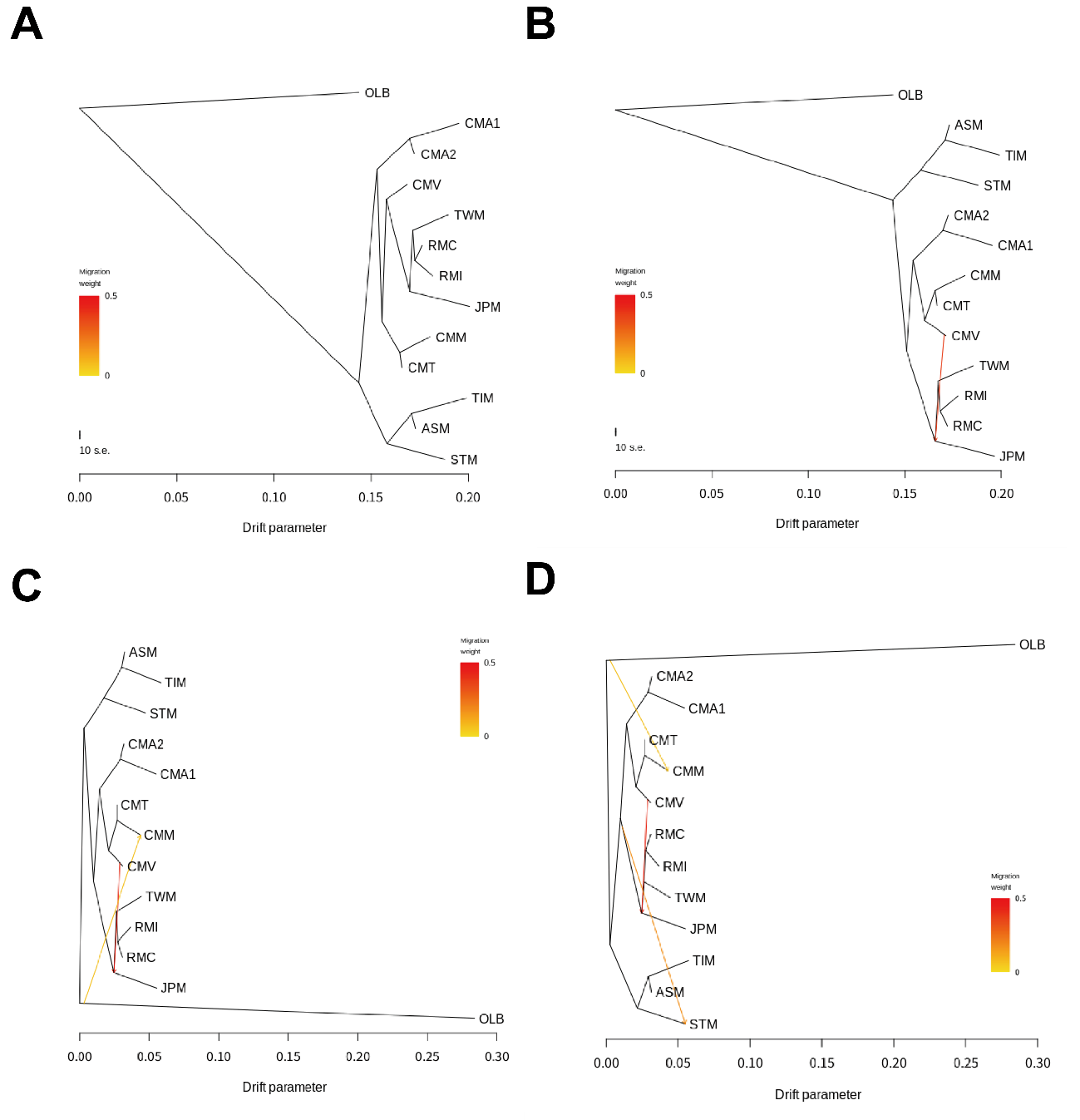
 **Supplementary Figure 3**

Population tree reconstructed using TreeMix with A) no migration, B) one migration edge, C) two migration edges, D) three migration edges. The color of migration edges represents the admixture fraction.

**
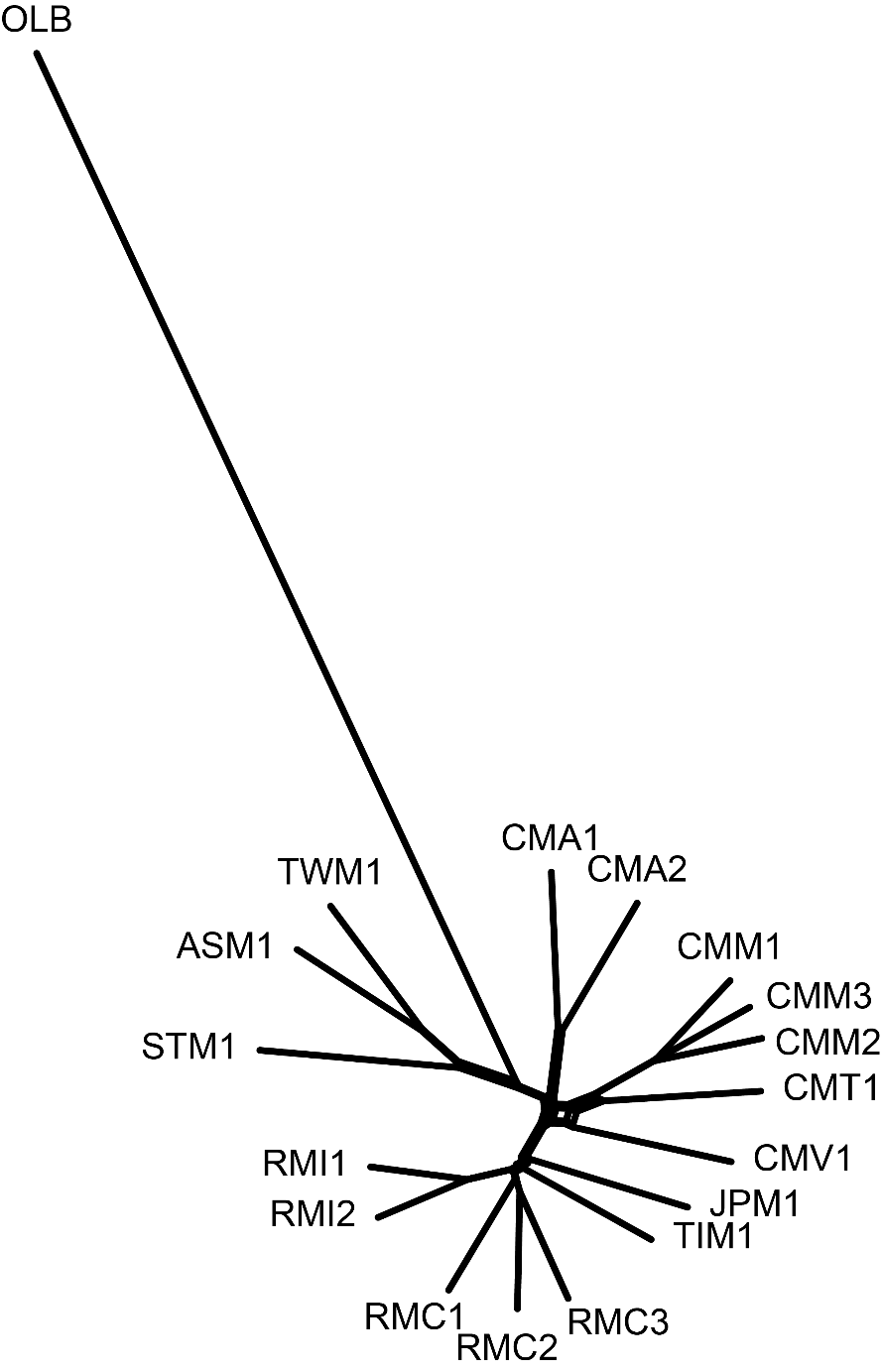
**

**Supplementary Figure 4**

Neighbor-net network. The names of samples are shown in Table 1 in the main text.


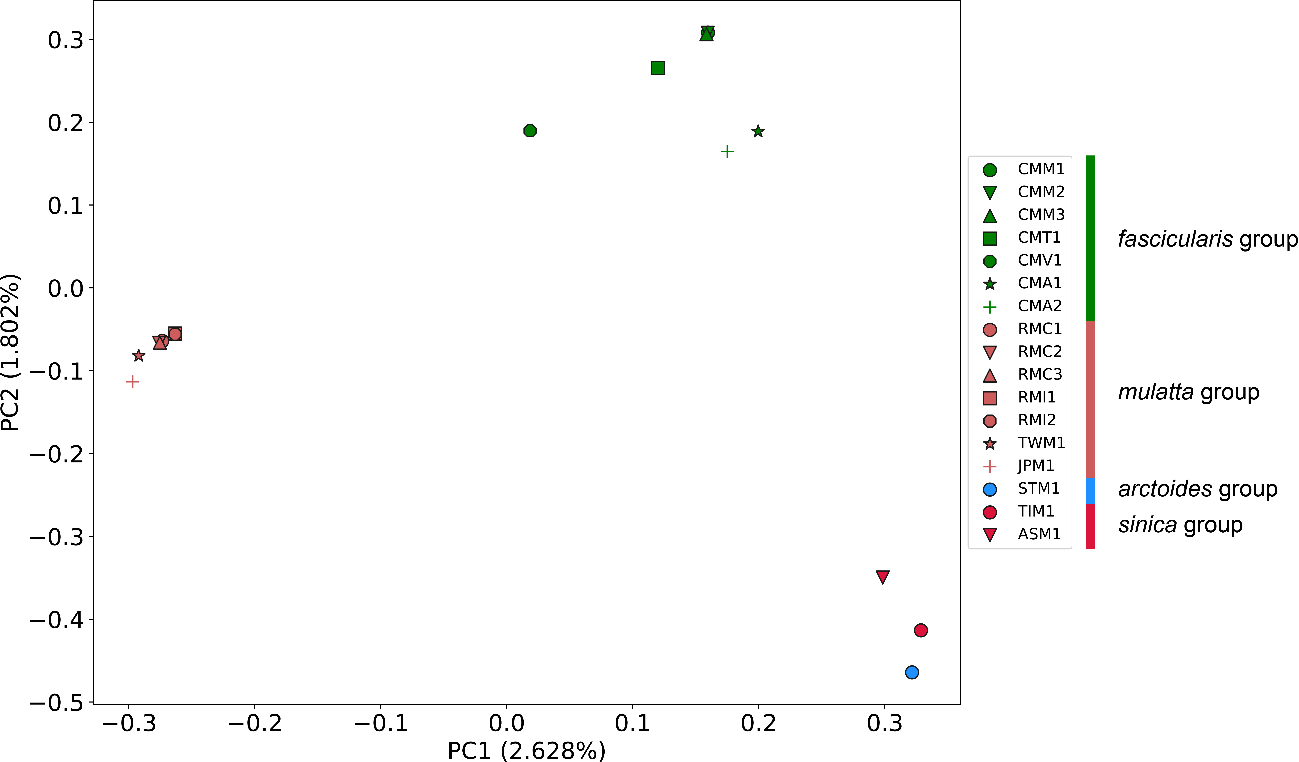


**Supplementary Figure 5**

The result of Principal component analysis (PCA) using autosomal data. The three major clusters correspond the *fascicularis* group, *mulatta* group, and *sinica*-*arctoides* group.


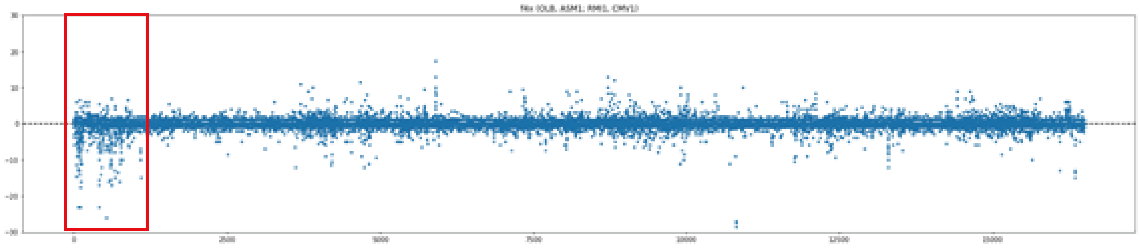


**Supplementary Figure 6**

Distribution of *f*4_X_(OLB, ASM; RMI, CMV) across the non-pseudo-autosomal region of the X chromosome. The *x*- and *y*-axes represent the order of SNV sorted by the genome coordinates and *f*4 values, respectively. The red rectangle region represents the 10 Mb region proximal to PAR1.


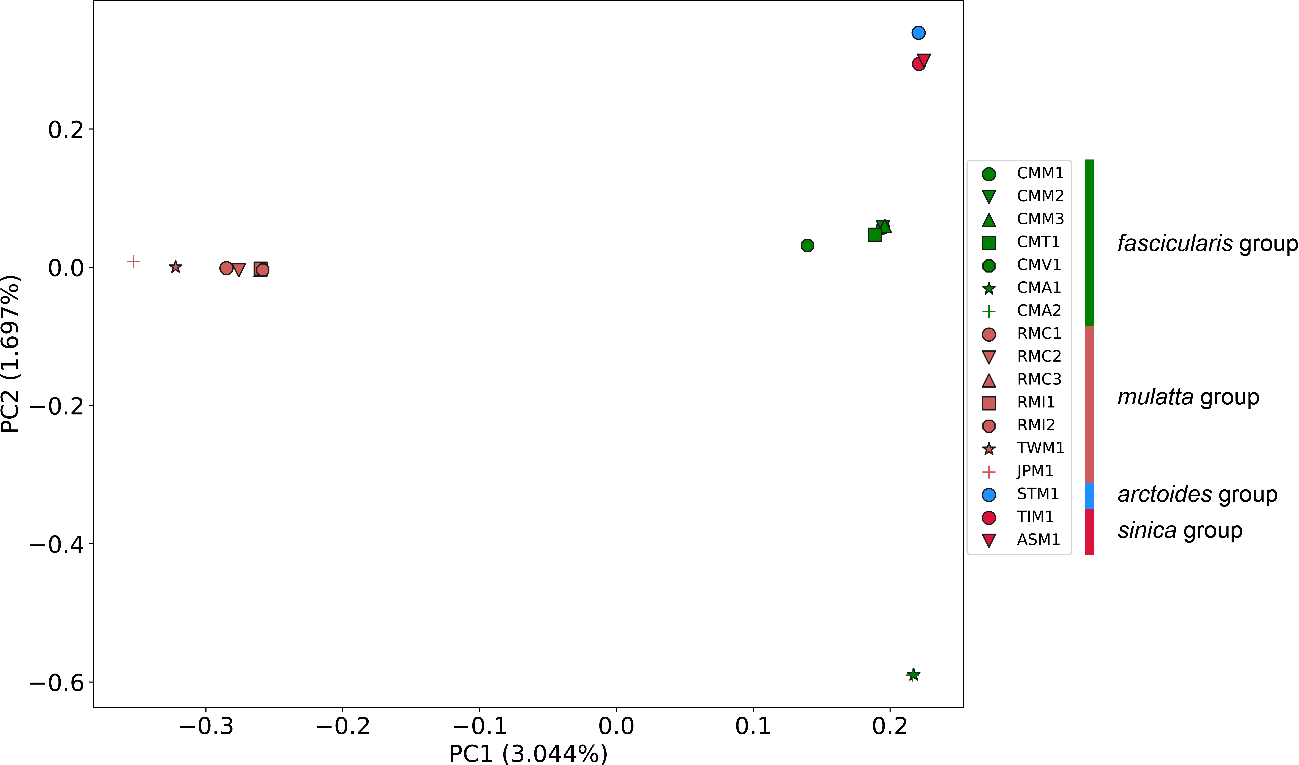


**Supplementary Figure 7**

The result of Principal component analysis (PCA) using X-chromosomal data. The 10 Mb regions adjacent to PAR1 was excluded from the analysis.

**A**


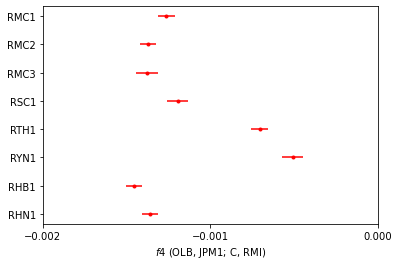


**B**


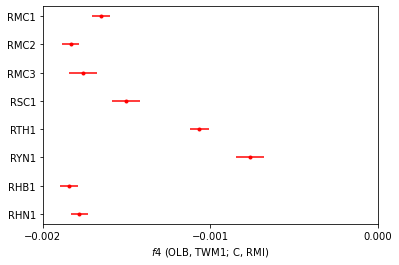


**Supplementary Figure 8**

The values of *f*4 statistics on autosomes. The names of the target species/population are shown on the left side of the panel. A) *f*4(OLB, JPM; C, RMI). Negative *f*4 statistics represent that JPM is more closely related to C than RMI. B) *f*4(OLB, TWM; C, RMI). Negative *f*4 represents that TWM is more closely related to C than RMI. RSC, *M. m. lasiotis*; RTH, *M. m. tcheliensis*; RYN, *M. m. mulatta*; RHB, *M. m. littoralis*, RHN, *M. m. brevicaudus*.


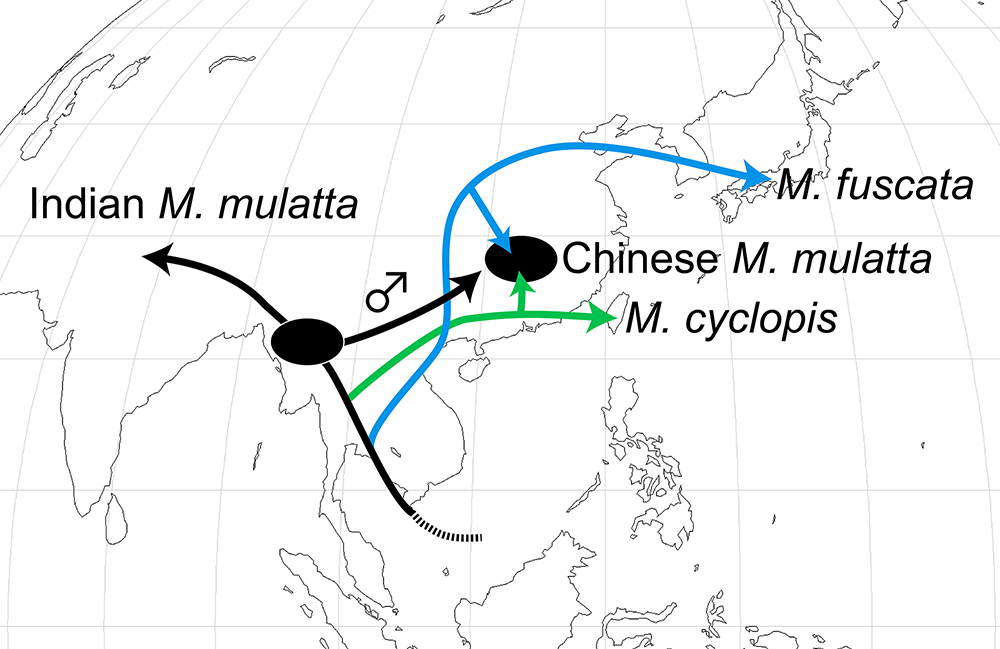


**Supplementary Figure 9**

A hypothetical evolutionary history of the *mulatta*-group species. Arrows indicate the direction of migration. We propose the existence of a ghost population generated by the admixture of populations diverged from the *M. fuscata* and *M. cyclopis* lineages. Strong male-biased migration from an ancestral Chinese *M. mulatta* population (represented by the symbol ♂) to the ghost population established the genetic features of extant Chinese *M. mulatta*. The gene flow between Chinese and Indian *M. mulatta* was detected in the previous study (Hernandez et al. 2007) but is omitted in this figure.
